# Isoflavones Isolated from the Seeds of *Millettia ferruginea* Induced Apoptotic Cell Death in Human Ovarian Cancer Cells

**DOI:** 10.3390/molecules25010207

**Published:** 2020-01-03

**Authors:** Yi-Yue Wang, Jun Hyeok Kwak, Kyung-Tae Lee, Tsegaye Deyou, Young Pyo Jang, Jung-Hye Choi

**Affiliations:** 1 Department of Life and Nanopharmaceutical Sciences, Kyung Hee University, Seoul 02447, Korea; yiyojanuary@naver.com (Y.-Y.W.); gjh2705@naver.com (J.H.K.); ktlee@khu.ac.kr (K.-T.L.); ypjang@khu.ac.kr (Y.P.J.); 2 Department of Chemistry, College of Natural Sciences, Salale University, Fitche, P.O. Box 245, Ethiopia; tsegdeyas@yahoo.com; 3 Department of Oriental Pharmaceutical Sciences, College of Pharmacy, Kyung Hee University, Seoul 02447, Korea

**Keywords:** *Millettia ferruginea*, 6,7-dimethoxy-3’,4’-methylenedioxy-8-(3,3-dimethylallyl) isoflavone, ferrugone, apoptosis, caspase, ovarian cancer

## Abstract

The seeds of *Millettia ferruginea* are used in fishing, pesticides, and folk medicine in Ethiopia. Here, the anti-cancer effects of isoflavones isolated from *M. ferruginea* were evaluated in human ovarian cancer cells. We found that isoflavone ferrugone and 6,7-dimethoxy-3’,4’-methylenedioxy-8-(3,3-dimethylallyl)isoflavone (DMI) had potent cytotoxic effects on human ovarian cancer cell A2780 and SKOV3. Ferrugone and DMI treatment increased the sub-G1 cell population in a dose-dependent manner in A2780 cells. The cytotoxic activity of ferrugone and DMI was associated with the induction of apoptosis, as shown by an increase in annexin V-positive cells. Z-VAD-fmk, a broad-spectrum caspase inhibitor, and z-DEVD-fmk, a caspase-3 inhibitor, significantly reversed both the ferrugone and DMI-induced apoptosis, suggesting that cell death stimulated by the isoflavones is mediated by caspase-3-dependent apoptosis. Additionally, ferrugone-induced apoptosis was found to be caspase-8-dependent, while DMI-induced apoptosis was caspase-9-dependent. Notably, DMI, but not ferrugone, increased the intracellular levels of reactive oxygen species (ROS), and antioxidant N-acetyl-L-cysteine (NAC) attenuated the pro-apoptotic activity of DMI. These data suggest that DMI induced apoptotic cell death through the intrinsic pathway via ROS production, while ferrugone stimulated the extrinsic pathway in human ovarian cancer cells.

## 1. Introduction

Ovarian cancer is the most lethal gynecological disease in the world. Approximately 225,000 women are diagnosed and about 140,200 women die each year from ovarian cancer [1]. Due to the non-specific symptoms, a majority of the patients are diagnosed when the disease is already advanced with poor prognosis [2]. The five-year survival rate of advanced ovarian cancer is only about 25%. Platinum-taxane chemotherapy following cytoreductive surgery is the standard treatment for ovarian cancer. Although primary therapy may be of therapeutic benefit in about 80% of ovarian cancer patients, around 70% of the patients eventually relapse [3]. Therefore, it is urgent to seek for new agents for the treatment of ovarian cancer. Natural products from plants have drawn great attention in this regard. Extensive studies have shown that plant-derived flavonoids have anti-cancer properties through the induction of apoptosis, which is usually abnormal in the cancer cells [4].

More than 200 species of the *Millettia* genus (Leguminosae) are distributed in the tropical and subtropical regions of Asia, Australia, and Africa [5]. In Africa, these plants are commonly used as insecticides and fish poison [6]. In addition, these plants have long been used in traditional medicine. For example, *Millettia pachycarpa* was used as an anthelminthic to evacuate the parasitic intestinal worms [7]. *M. thonningii* is known to treat dysentery, constipation, and diarrhea while *M. carulea* has anti-inflammatory effects [8,9]. *M. macrophylla* is also used to relieve menopausal symptoms [10].

Phytochemical studies with *Millettia* species revealed that these species have a rich source of flavonoids, including isoflavonoids, rotenoids, chalcones, and pterocarpans [11,12]. *M. ferruginea* (Hochst) Baker, is an endemic of Ethiopia and has two representative species; ssp. *ferruginea* and *darassana*. Previously, from the seeds of *M. ferruginea* ssp. *ferruginea,* we isolated several isoflavones, including calopogonium isoflavone A, durmillone, barbigerone, ferrugone, prebarbigerone, and a novel prenylated isoflavone 6,7-dimethoxy-3’,4’-methylenedioxy-8-(3,3-dimethylallyl)isoflavone (DMI) [12]. Calopogonium isoflavone A and durmillone showed antimicrobial and DNA fragmentation effects against gram-negative bacteria and the promastigotes of *Leishmania donovani* [13], while barbigerone exhibited inhibitory effects on the growth and metastasis of tumors in melanoma [14]. However, to the best of our knowledge, the biological activities of ferrugone, prebarbigerone, and DMI have never been reported. Here, we investigated the anti-cancer effects of the isoflavones using human ovarian cancer cells.

## 2. Results

### 2.1. Ferrugone and DMI Isolated from M. ferruginea Had Potent Cytotoxic Activities against Human Ovarian Cancer Cells

We investigated the effects of the three isoflavones isolated from *M. ferruginea,* ferrugone, prebarbigerone, and DMI on the viability of two human ovarian cancer cells A2780 and SKOV3. Ferrugone and DMI had potent cytotoxic activities in A2780 cells (Table 1).

**Figure 1:**
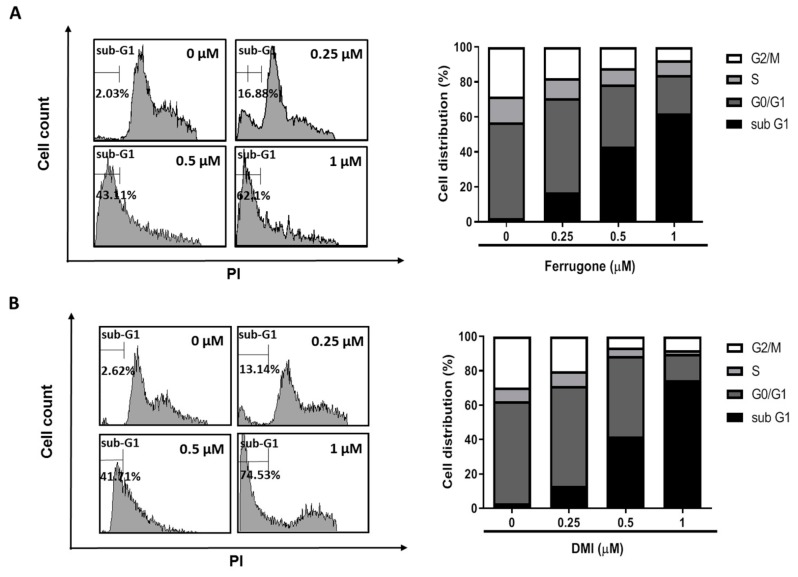
Effect of ferrugone and DMI on cell cycle distribution in human ovarian cancer cells. A2780 cells were treated with ferrugone (**A**) or DMI (**B**) (0.25, 0.5, and 1 µM) for 48 h and then stained with propidium iodide (PI). The cell-cycle distribution profiles of the cells were determined by flow cytometry. The graph indicates the percentages of cells in the sub-G1, G0/G1, S, and G2/M phases of the cell cycle. The data are representative of three independent experiments.

To examine whether the cytotoxic activities of ferrugone and DMI are related to cell cycle arrest, the distribution of the cells in different phases of cell cycle progression was analyzed in A2780 cells. As shown in Figure 1, treatment with ferrugone or DMI increased sub G1 phase in the ovarian cancer cells. After treatment with 0, 0.25, 0.5, and 1 µM of ferrugone, the proportions of the sub G1 phase cells were 2.03%, 16.88%, 43.11%, and 62.1% (Figure 1A). Following DMI treatment under the same condition, the proportions of the sub G1 cells were 2.62%, 13.14%, 41.71%, and 74.53% (Figure 1B). These data suggest that the cytotoxicities of ferrugone and DMI are mediated by the induction of cell death rather than cell cycle arrest.

### 2.2. Ferrugone and DMI Isolated from M. ferruginea Induced Apoptotic Cell Death in the Ovarian Cancer Cells 

**Figure 2:**
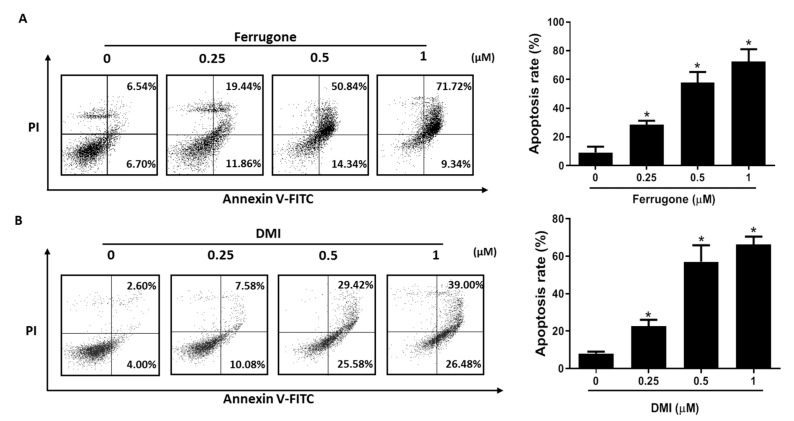
Effect of ferrugone and DMI on apoptotic cell death in human ovarian cancer cells. A2780 cells were treated with ferrugone (**A**) and DMI (**B**) (0.25, 0.5, and 1 µM) for 48 h and co-stained with PI and Annexin V-FITC (fluorescein isothiocyanate). The translocation of phosphatidyl serine was detected by flow cytometry. The graph indicates the percentages of annexin V-positive apoptotic cells in the right quadrants of flow cytometry graphs. The data are representative of three independent experiments. Data were analyzed using one-way ANOVA followed by Dunnett’s multiple comparison test. * *p* < 0.05 as compared with untreated group.

We further investigated whether the cytotoxic effects of ferrugone and DMI were related to apoptotic cell death using annexin V-FITC (fluorescein isothiocyanate) staining assay. As shown in Figure 2, both ferrugone and DMI increased annexin V-positive apoptotic cells in the right quadrants of the flow cytometry graphs. Treatment with 0.25, 0.5, and 1 μM of ferrugone induced a dose-dependent increase in the apoptotic cell population from 13.24% to 31.3%, 65.18%, and 81.06%, respectively (Figure 2A). Treatment with 0.25, 0.5, and 1 μM of DMI showed 17.66%, 55%, and 65.48% apoptotic cell death, respectively (Figure 2B). These results suggest that ferrugone and DMI dose-dependently induced apoptotic cell death in human ovarian cancer cells.

### 2.3. Ferrugone and DMI Induced Caspase-Dependent Apoptotic Cell Death in the Ovarian Cancer Cells

**Figure 3:**
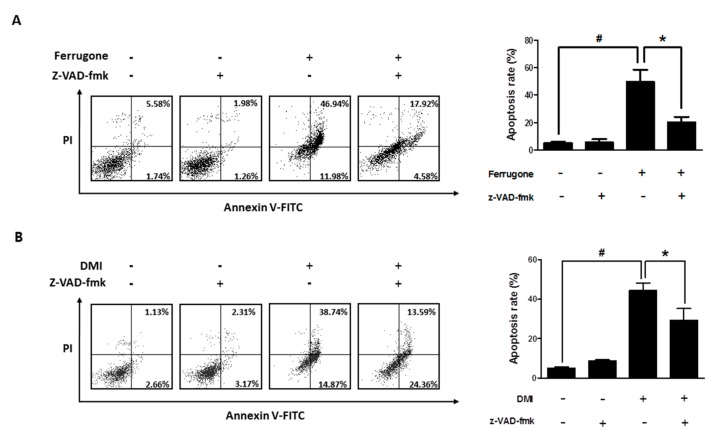
Involvement of caspases in ferrugone and DMI-induced cell death in human ovarian cancer cells. A2780 cells were pretreated with broad caspase inhibitor z-VAD-fmk (50 µM) for 2 h, and then treated with ferrugone (**A**) and DMI (**B**) (0.5 µM) for 48 h. Annexin/PI V-FITC staining assay was performed to determine apoptosis. The graph indicates the percentages of annexin V-positive apoptotic cells in the right quadrants of flow cytometry graphs. The data are representative of three independent experiments. Student’s *t*-test (two-tailed) was applied to evaluate the significance. # *p* < 0.05 as compared with the untreated group. * *p* < 0.05 as compared with the ferrugone or DMI only-treated group.

**Figure 4:**
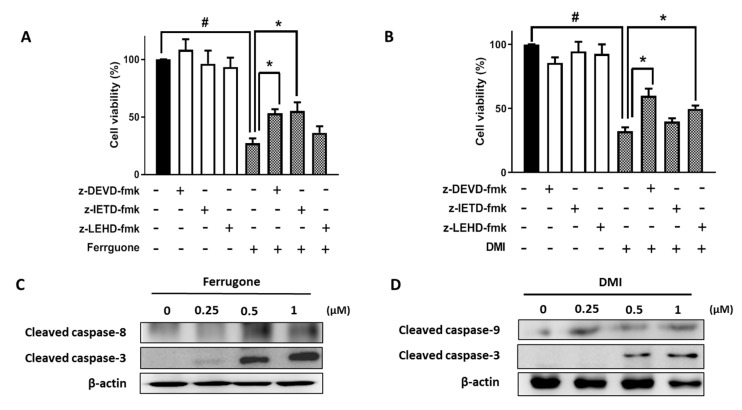
Involvement of caspase-3, -8, and -9 in ferrugone and DMI-induced cell death in human ovarian cancer cells. (**A**,**B**) A2780 cells were pretreated with caspase-3 inhibitor z-DEVD-fmk (75 µM), caspase-8 inhibitor z-IETD-fmk (50 µM), and caspase-9 inhibitor z-LEHD-fmk (75 µM) for 2 h, and then treated with ferrugone (**A**) and DMI (**B**) (0.5 µM) for 24 h. MTT assay was performed to determine cell death. (**C**,**D**) A2780 cells were treated with ferrugone (**C**) and DMI (**B**) (0.25, 0.5, and 1 µM) for 48 h. Cleaved caspase-3 and caspase-8 levels were determined by Western blot assay. β-actin was used as an internal control. The data are representative of three independent experiments. Student’s *t*-test (two-tailed) was applied to evaluate the significance. # *p* < 0.05 as compared with the untreated group. * *p* < 0.05 as compared with the ferrugone or DMI only-treated group.

The involvement of caspases in apoptosis induced by ferrugone and DMI was further investigated considering the critical role of various caspases in apoptotic cell death. A broad caspase inhibitor, z-VAD-fmk, significantly suppressed the ferrugone- and DMI-induced apoptotic cell death in the A2780 cells, suggesting that both ferrugone and DMI induced caspase-dependent apoptosis in the human ovarian cancer cells (Figure 3A,B). To confirm which caspase was associated in particular, z-DEVD-fmk (caspase-3 inhibitor), z-IETD-fmk (caspase-8 inhibitor), and z-LEHD-fmk (caspase-9 inhibitor) were used (Figure 4). z-DEVD-fmk and z-IETD-fmk, but not z-LEHD, significantly decreased ferrugone-induced cell death (Figure 4A). On the other hand, DMI-induced cell death was significantly reversed by z-DEVD-fmk and z-LEHD-fmk, but not z-IETD-fmk (Figure 4B). Additionally, western blot analysis revealed that ferrugone activated caspase-3 and caspase-8 (Figure 4C) and DMI activated caspases-3 and caspase-9 (Figure 4D) in the A2780 cells resulting in increasing the density of their cleaved forms. These data suggest that ferrugone and DMI induced caspase-3-dependent apoptosis via the caspase-8 and caspase-9 activation, respectively, in the human ovarian cancer cells.

### 2.4. Apoptosis-Inducing Effect of DMI Is Associated with ROS Production

**Figure 5:**
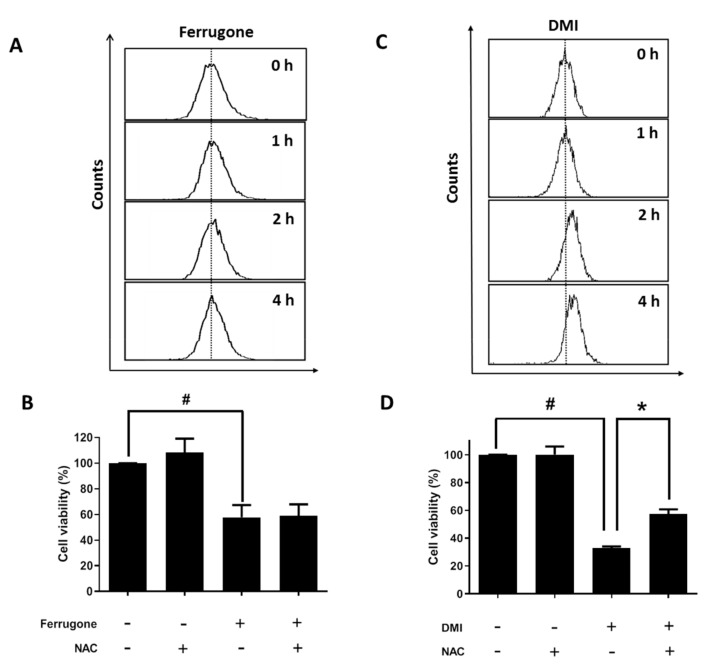
Involvement of ROS (reactive oxygen species) in DMI-induced cell death in human ovarian cancer cells. (**A**) A2780 cells were treated with 0.5 µM of ferrugone for the indicated times (1, 2, and 4 h). The cells were stained with DCF-DA (2’,7’–dichlorofluorescin diacetate), and analyzed by flow cytometry. (**B**) A2780 cells were pretreated with N-acetyl-L-cysteine (NAC, 5 mM) for 30 min, then treated with ferrugone (0.5 µM) for 48 h. MTT assay was performed to examine the cell death. (**C**) A2780 cells were treated with 0.5 µM of DMI for the indicated times (1, 2, and 4 h). The cells were stained with DCF-DA and analyzed by flow cytometry. (**D**) A2780 cells were pretreated with NAC (5 mM) for 30 min, then treated with DMI (0.5 µM) for 48 h. MTT assay was performed to examine the cell death. Student’s *t*-test (two-tailed) was applied to evaluate the significance. # *p* < 0.05 as compared with the untreated group. * *p* < 0.05 as compared with the ferrugone or DMI only treated group.

Reactive oxygen species (ROS) are known to stimulate apoptosis in various cancer cells [9]. Here, we investigated the involvement of ROS in ferrugone or DMI-induced cell death in human ovarian cancer. DCF-DA (2’,7’–dichlorofluorescin diacetate) staining assay showed that ferrugone did not induce any significant change in the intracellular ROS level (Figure 5A), and N-acetyl-L-cysteine (NAC) treatment did not affect ferrugone-induced cell death in A2780 cells (Figure 5B). On the other hand, DMI increased the intracellular ROS level in A2780 cells (Figure 5C). Additionally, DMI-induced cell death was significantly inhibited in the presence of N-acetyl-L-cysteine (NAC) (Figure 5D). These data reveal that DMI, but not ferrugone, induced apoptosis by ROS regulation in the ovarian cancer cells.

## 3. Discussion

The genus *Millettia* appears in the African pharmacopeia. It has a wide range of biological activities as confirmed by pharmacological studies, such as pesticidal, antiviral, anti-inflammatory, antitumoral, and bactericidal activities, and therefore, this genus has attracted great attention in traditional medicine and research of new biologically active compounds [6]. In addition, the genus *Millettia* is rich in flavonoids, for example, isoflavones, showing various activities in women health. Isoflavones have been shown to have beneficial effects for women with menopausal complaints, postmenopausal syndromes, and breast and gynecologic cancers [15,16,17,18]. Similarly, griffonianone C, an isoflavone from the *M. griffoniana,* has been reported to have estrogenic activity in uterus and liver of ovariectomized rats [19]. In addition, isoflavones extracted from *M. taiwaniana* have shown cancer chemopreventive activities [20]. Durmillone isolated from *M. pachyloba* Drake induced apoptosis and autophagy in Hela and MCF-7 cells [21]. In this study, we demonstrated that two isoflavones, ferrugone and DMI, isolated from *M. ferruginea*, induced apoptotic cell death in human ovarian cancer cells.

In this study, to investigate the effects of isoflavones on the viability of human ovarian cancer cells, we have used two most commonly used human ovarian epithelial cancer cell lines A2780 and SKOV3 cells. Both DMI and ferrugone exhibited more potent cytotoxicity on A2780 cells with an observed IC_50_ value of 0.44 ± 17.24 and 0.51 ± 0.06 μM, respectively, than cisplatin (IC_50_ value of 14.14 ± 0.59 μM). However, in SKOV3 cells, ferrugone showed only mild cytotoxic effect (IC_50_ value of 36.78 ± 5.26 μM) and DMI was not active (IC50 value >100 uM). These data indicate that DMI and ferrugone have potent cytotoxicity only in A2780 cells, but not in SKOV3 cells. Notably, A2780 cells express wild-type p53 gene while SKOV3 cells are p53 null, suggesting that the isoflavone-induced cell death might be p53 dependent. It remains to be further demonstrated whether p53 mediate DMI- and ferrugone-induced apoptosis in human ovarian cancer cells.

Apoptosis is a natural mechanism of programmed cell death, which plays a critical role in the development and homeostasis of mammals that live long [22]. The prevention of apoptosis is an eminent hallmark of cancer, which may not only promote tumorigenesis but also render the cancer cells resistant to treatment [23,24]. Thus, apoptosis is a promising target for cancer therapy. The protease enzymes caspases (cysteinyl aspartate-specific protease) play critical roles in programmed cell death, as well as inflammation. Based on their function, mammalian caspase-2, -3, -7, -8, -9, and -10 are apoptotic caspases, whereas caspase-1, -4, -5, -11, and -12 are involved in inflammation [25]. The upstream signaling events induce dimerization and activation of initiator caspases-8 and -9, and then, the effector caspases-3, -6, and -7 are cleaved by initiator caspases resulting in apoptosis [26]. There are two common pathways that can be activated by caspase activation. In the extrinsic pathway, caspase-8 activation by death ligands binds to a death receptor in response to extracellular signals, while the intracellular stimulants activate caspase-9 in the intrinsic pathway [27]. The intrinsic and extrinsic pathways converge to caspase-3, which cleaves the inhibitor of the caspase-activated deoxyribonuclease, leading to apoptosis [28]. In this study, we found that both ferrugone and DMI induced the activation of an effector caspase-3 and caspases-dependent apoptosis in human ovarian cancer cells. Notably, ferrugone induced the activation of caspase-8, but not caspase-9, while DMI stimulated the activation of caspase-9, but not caspase-8. These data suggested that ferrugone and DMI differently induced apoptosis via a caspase-8-dependent extrinsic pathway and caspase-9-dependent intrinsic pathway, respectively.

The accumulation of reactive oxygen species (ROS) is involved in multiple diseases, including atherosclerosis, neurodegeneration, diabetes, aging, and cancer [29]. In cancer therapy, treatment with drugs that induce ROS generation has been suggested as an effective strategy to selectively push cancer cells over the ROS threshold and into cell death [30]. Intracellular ROS are known to activate the intrinsic apoptosis pathway through the reduction of mitochondria membrane potential, release of death-promoting factors, such as cytochrome c from the mitochondria, and the activation of caspase-9 [31,32]. Additionally, it has been suggested that oxidative modification of caspase-9 by ROS induces the interaction with apoptotic protease-activating factor-1 (Apaf-1), resulting in the stimulation of its auto-cleavage and activation [33]. In this study, we demonstrated that DMI increased the intracellular production of ROS. More importantly, caspase-9-dependent apoptotic cell death by DMI was significantly reversed by antioxidant NAC treatment. These data suggest that DMI-induced ROS production may stimulate caspase-9 activation, regulating apoptotic cell death. Although many polyphenols including isoflavones have antioxidant activity and show cytoprotective effect in some cells by reducing the ROS levels, some polyphenols have been reported to act both as an antioxidant and as a pro-oxidant. For instance, several polyphenols, including isoflavones and phlorotannins, increase intracellular levels of ROS to induce apoptosis and cell death in cancer cells [34,35]. That is consistent with our finding showing the isoflavone DMI induced ROS production and apoptosis in human ovarian cancer cells. In addition, increased levels of ROS have been reported to stimulate apoptotic cell death in cancer cells by regulating the stress kinase pathways, including PI3K/Akt and MAPK, as well as caspase pathway. Thus, whether those stress kinase pathways are involved in the DMI-induced ROS production and apoptosis should be further investigated. On the other hand, it is of note that the activation of the extrinsic apoptosis pathway by another isoflavone, ferrugone, was not associated with ROS production in human ovarian cancer cells. Further research is required to illuminate the upstream molecular mechanisms underlying ferrugone-induced apoptotic cell death.

Here, we found that the two isoflavones, ferrugone and DMI, induced apoptosis through a different pathway in human ovarian cancer cells. The structural differences between two isoflavones are that ferrugone has two methoxy groups at 2, 5 positions of the B ring; and a methoxy group and an isoprene unit at 7, 8 positions of A ring constitutes a new ring and demethoxylation at 6 position of A ring. The difference in the mechanisms induced by ferrugone and DMI might be explicated by these structural differences, which potentially lead to the binding of different target molecules. Further research is required to clarify the detailed molecular mechanisms underlying the structure-activity relationship.

## 4. Materials and Methods 

### 4.1. Sample Preparation

The mature seeds of *Millettia ferruginea* ssp. *ferruginea* were collected from Oromia region, Bacho Faleni district of rural lowland area, northern part of Ethiopia. The collected plant material was identified by one author (Deyou T), and a voucher specimen (TD-02/2016) was deposited at Jimma University Herbarium, Ethiopia. The phytochemical study of 6,7-dimethoxy-3’,4’-methylenedioxy-8-(3,3-dimethylallyl) isoflavone, ferrugone, and prebarbigerone (Table 1) used in the present study was described in our previous study [12]. Briefly, dry mature seeds of *M. ferruginea* ssp. *ferruginea* (300 g) were powdered and extracted with CH_2_Cl_2_/CH_3_OH (1:1, v/v). Resulting crude extract (160 g) was subjected to liquid–liquid extraction in CH_3_OH/*n*-hexane (7:3) to give 110 g of CH_3_OH extract. Successive chromatographic separation of CH_3_OH extract gave three prenylated isoflavone compounds, 6,7-dimethoxy-3’,4’-methylenedioxy-8-(3,3-dimethylallyl) isoflavone (34.6 mg), ferrugone (80.9 mg), and prebarbigerone (131.4 mg). The stock solution of each compound was prepared in DMSO (dimethyl sulfoxide) and serially diluted to set optimal concentration for bioassay. The final concentration of DMSO in the experiments was less than 0.1%.

### 4.2. Materials

Roswell Park Memorial Institute (RPMI) 1640, penicillin, fetal bovine serum (FBS), and streptomycin were purchased from Life Technologies Inc. (Grand Island, NY, USA). 3-(4,5-Dimethylthiazol-2-yl)-2,5-diphenyl tetrazolium bromide (MTT) was obtained from Molecular Probes Inc. (Eugene, OR, USA). Propidium iodide (PI), N-acetyl-L-cysteine (NAC), and 2-mercaptoethanol were acquired from Sigma Chemical (St. Louis, MO, USA). Phenylmethylsulfonylfluoride (PMSF) and annexin V-fluorescein isothiocyanate (FITC) were purchased from BD Biosciences (San Jose, CA, USA). Dichlorofluorescein diacetate (DCFH-DA) was obtained from Santa Cruz Biotechnology (Santa Cruz, CA, USA). All inhibitors for caspases were purchased from Calbiochem (Bad Soden, Germany). Enhanced chemiluminescence (ECL) reagent was obtained from EMD Millipore (Billerica, MA, USA). Tris-buffered saline was obtained from Boster Biological Technology Ltd. (Wuhan, China). Caspase-3 and caspase-9 antibodies were procured from Cell Signaling Technology (Beverly, MA, USA). Caspase-8 and β-actin antibodies were procured from Santa Cruz Biotechnology (Santa Cruz, CA, USA). Secondary antibodies were purchased from The Jackson Laboratory (West Grove, PA, USA).

### 4.3. Cell Culture

Ovarian cancer cells (A2780 and SKOV3) were cultured in RPMI 1640 supplemented with 5% FBS, penicillin (100 U/mL), and streptomycin sulfate (100 μg/mL). The cells were incubated in a 5% CO_2_ and 95% air humidified atmosphere at 37 °C.

### 4.4. MTT Assay

Cell viability was examined using the MTT assay. Human ovarian cancer cells (A2780 and SKOV3) were seeded in a 96-well plate containing 50 μL of RPMI medium at a density of 1 × 10^5^ cells/mL in each well. After 24 h incubation, various concentrations of ferrugone and DMI dissolved in dimethyl sulfoxide were diluted with culture medium and were added into each well. After 48 h incubation, 50 μL of MTT (1 mg/mL stock solution) was added, and then the plates were incubated for an additional 4 h. Then the medium was discarded, and the formazan crystal that formed in the cells was dissolved in 50 μL of DMSO in each well. The optical density was measured at 540 nm by using a microplate spectrophotometer (SpectraMax; Molecular Devices, Sunnyvale, CA, USA).

### 4.5. Propidium Iodide (PI) Staining for Cell Cycle Analysis

A2780 cells were seeded in 60 mm culture dishes containing 3 mL of RPMI medium at a density of 1 × 10^5^ cells/mL in each dish. After 24 h incubation, the cells were treated with ferrugone and DMI for 48 h and collected in ice-cold phosphate-buffered saline (PBS). After being washed twice with cold PBS, the cells were fixed with EtOH (70%) and stored at 4 °C for 2 h. Fixed cells were suspended in a PI staining solution (50 μg/mL) supplemented with RNase A (250 μg/mL). The suspended cells were incubated in the dark at room temperature for 20 min. The fluorescent intensity of the cells was measured using a Guava easyCyte flow cytometry system (EMD Milipore, Bilerica, MA, USA).

### 4.6. Annexin V and PI Double Staining for Apoptosis Analysis

A2780 cells were seeded in 60 mm culture dishes containing 3 mL of RPMI medium at a density of 1 × 10^5^ cells/mL in each dish. After 24 h incubation, the cells were treated with ferrugone and DMI for 48 h. The cells were harvested and washed twice with cold PBS and suspended with 500 μL of binding buffer (10 mM HEPES (4-(2-hydroxyethyl)piperazine-1-ethanesulfonic acid)/NaOH, 140 mM NaCl, 2.5 mM CaCl_2_, PH 7.4). After staining with 1.25 μL of FITC-conjugated Annexin V for 15 min and 10 μL of PI (50 mg/mL) for 5 min in the dark place, the mixture was analyzed by Guava^®^ easyCyte flow cytometry.

### 4.7. Western Blot Analysis

A2780 cells were seeded in 60mm culture dishes containing 3 mL of RPMI medium at a density of 1 × 10^5^ cells/mL in each dish. After 24 h incubation, the cells were treated with ferrugone and DMI for 48 h. The cells were collected and washed twice with cold PBS. Total cellular proteins were extracted using protein lysis buffer (Intron Biotechnology, Seoul, Korea) following manufacturer’s instructions and protein concentrations were measured by the Bradford assay. The protein extracts were mixed with 5x SDS (sodium dodecyl sulfate) sample buffer and heated for 5 min at 95 °C. The mixture was loaded on a gel for SDS (sodium dodecyl sulfate)-PAGE (polyacrylamide gel electrophoresis). After electrophoretic separation, separated proteins were blotted to PVDF (polyvinylidene difluoride) membranes. After blocking with 5 % skim milk for 1 h, the membranes were incubated overnight at 4 °C with diluted primary antibodies against caspase-3, -8, 9, and β-actin in TBS-T (Tris-buffered saline containing Tween-20). After a subsequent washing three times with TBS-T, the membranes were incubated with an appropriate secondary antibody at room temperature for 2 h. Immunoreactive bands were visualized by the ECL kit and detected by ImageQuant Las-4000 (GE Healthcare Life Science, WI, USA).

### 4.8. Measurement of Reactive Oxygen Species

The intracellular levels of ROS were measured by using the fluorescent probe DCFH-DA that is commonly used to measure H_2_O_2_. A2780 cells were seeded in 60 mm culture dishes containing 3 mL of RPMI medium at a density of 1 × 10^5^ cells/mL in each dish. After 24 h incubation, the cells were collected by centrifugation after treatment with ferrugone and DMI at the desired time intervals, resuspended in PBS, and loaded with 20 μM DCFH-DA. The fluorescent intensity was analyzed by flow cytometry.

### 4.9. Statistical Analysis

One-way ANOVA or student’s *t*-tests were performed to identify statistically significant differences. *p* values less than 0.05 were considered statistically significant.

## 5. Conclusions

These data suggest that DMI induced apoptotic cell death through the intrinsic pathway via ROS production, while ferrugone stimulated the extrinsic pathway in human ovarian cancer cells. This is the first study to demonstrate the biological activity of DMI and ferrugone.

## Figures and Tables

**Table 1:**
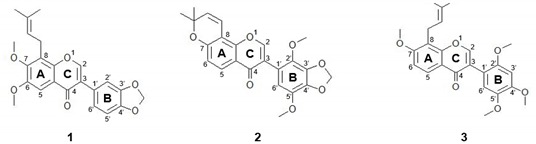
Cytotoxic activity of isoflavones isolated from *Millettia ferruginea* spp. *ferruginea* in human ovarian cancer cells.

Compound No	Name	IC_50_^*a*^	
		A2780	SKOV3
**1**	6,7-dimethoxy-3′,4′-methylenedioxy-8- (3,3-dimethylallyl)isoflavone (DMI)	0.45 ± 0.06 μM (0.18 ± 0.02 μg/mL)	> 100 μM (> 39.41 μg/mL)
**2**	Ferrugone	0.51 ± 0.06 μM (0.21 ± 0.02 μg/mL)	36.78 ± 5.26 μM (15.01 ± 2.15 μg/mL)
**3**	Prebarbigerone	32.06 ± 8.31 μM (13.16 ± 3.41 μg/mL)	> 100 μM (> 41.05 μg/mL)
**-**	Cisplatin *^b^*	14.14 ± 0.59 μM (4.24 ± 0.18 μg/mL)	36.64 ± 0.96 μM (10.99 ± 0.29 μg/mL)

*^a^* IC_50_ values are defined as the concentration that results in a 50% decrease in the number of cells compared with that of the control cultures. *^b^* Cisplatin was used as a positive control.
